# Vascular Effects on Cerebrovascular Permeability and Neurodegeneration

**DOI:** 10.3390/biom13040648

**Published:** 2023-04-04

**Authors:** Nurul Sulimai, Jason Brown, David Lominadze

**Affiliations:** 1 Department of Surgery, College of Medicine, University of South Florida Morsani, Tampa, FL 33612, USA; nurulsulimai@usf.edu (N.S.); jasonb3@usf.edu (J.B.); 2 Department of Molecular Pharmacology and Physiology, College of Medicine, University of South Florida Morsani, Tampa, FL 33612, USA

**Keywords:** blood-brain-barrier, blood proteins, fibrinogen and cognitive impairment

## Abstract

Neurons and glial cells in the brain are protected by the blood brain barrier (BBB). The local regulation of blood flow is determined by neurons and signal conducting cells called astrocytes. Although alterations in neurons and glial cells affect the function of neurons, the majority of effects are coming from other cells and organs of the body. Although it seems obvious that effects beginning in brain vasculature would play an important role in the development of various neuroinflammatory and neurodegenerative pathologies, significant interest has only been directed to the possible mechanisms involved in the development of vascular cognitive impairment and dementia (VCID) for the last decade. Presently, the National Institute of Neurological Disorders and Stroke applies considerable attention toward research related to VCID and vascular impairments during Alzheimer’s disease. Thus, any changes in cerebral vessels, such as in blood flow, thrombogenesis, permeability, or others, which affect the proper vasculo-neuronal connection and interaction and result in neuronal degeneration that leads to memory decline should be considered as a subject of investigation under the VCID category. Out of several vascular effects that can trigger neurodegeneration, changes in cerebrovascular permeability seem to result in the most devastating effects. The present review emphasizes the importance of changes in the BBB and possible mechanisms primarily involving fibrinogen in the development and/or progression of neuroinflammatory and neurodegenerative diseases resulting in memory decline.

## 1. Introduction

During this past decade, greater emphasis in neuroscience has been given to problems associated with vascular cognitive impairment and dementia (VCID) [1]. As a result, out of 132 Research Priorities that have been used as a guide by the National Health, Lung, and Blood Institute for this last decade, two compelling questions relate to vascular effects on neurodegeneration. They are “What interdependencies between the brain/peripheral nervous system are important to the development, progression, manifestations, and treatment of cardiac and vascular disease?” and “What pathobiology underlies vascular causes of cognitive decline?” [2]. Inflammation can be one of the main causes in the development of VCID.

Research has shown a strong link between cardiovascular diseases, along with cerebrovascular diseases, and subsequent cognitive impairment and dementia [3]. During some neuroinflammatory diseases, VCID is often presented as a co-morbidity. For example, Alzheimer’s disease (AD) is the leading cause of dementia, and it is often accompanied by VCID. It is estimated that 40% of AD patients also have some form of VCID. Vascular dementia accounts for about 15–30% of dementia cases worldwide [3,4]. Other diseases that are known to cause a substantial cognitive impairment are traumatic brain injury (TBI) [5,6,7,8] and multiple sclerosis (MS) [9]. Although dementia is not the primary clinical sign associated with stroke, stroke almost doubles the risk of developing dementia later in life [10]. The risk of dementia in stroke patients after the incident depends on the lesion size and location, but stroke survivors also suffer worsened cognition years later for reasons not well understood [10]. While incidence of dementia can be close to 5% after transient ischemic stroke, its occurrence can reach 34% after severe stroke [3,4]. Ischemic stroke is the most common type of stroke, making up 87% of all strokes where brain ischemia causes substantial neuronal damage. It has been shown that aggravated peripheral inflammatory response to stroke caused by preceding systemic inflammation has deleterious actions on components of the neurovascular unit (NVU) that may affect BBB integrity [11]. Some common mechanisms associated with cerebrovascular-driven cognitive impairment are associated with accumulation of abnormal proteins, oxidative stress, early synaptic disconnection, and apoptosis leading to cell death. All these abnormalities can be positively accompanied by alterations in the blood brain barrier (BBB). There are many studies that link BBB dysfunction with dementia in humans [12,13,14,15] and in animal models [14,15,16,17]. The objective of this review is to underline the importance of changes in the cerebrovascular permeability resulting in accumulation of blood plasma proteins and particularly of fibrinogen (Fg) in the extravascular space of the brain, as well as to discuss some possible mechanisms involved in the development and/or progression of neuroinflammatory and neurodegenerative diseases resulting in memory decline. 

## 2. Inflammation and Thrombogenesis 

Inflammation is one the most important factors that cause changes in normal homeostasis in the body. Many neurodegenerative diseases are associated with inflammation and considered neuroinflammatory diseases. For example, TBI [18,19,20], AD [21], MS [22,23], and stroke [24] are considered neuroinflammatory diseases. The main components of the circulatory system that can be affected by inflammation and may result in neurodegeneration are blood cells, such as platelets, leukocytes, and erythrocytes, and vascular wall components, such as endothelial cells (ECs), smooth muscle cells, and pericytes. For example, blood samples from AD patients showed an increased number of activated platelets compared to that in samples from the control group [25]. Platelets, major players in hemostasis and thrombosis [21], have also been known to have a significant effect during inflammation [26]. Activation of platelets and their increased aggregation have been documented during neurodegenerative diseases such as TBI [27], AD [25], and MS [28]. It has been shown that AD mutations result in a significantly hyperactivated state of circulating platelets where the platelets from 3XTg-AD mice adhere more avidly on matrices and have an increased ability to form thrombi during normal flow condition [29]. Therefore, it is well-accepted that platelets are not only activated as a result of inflammation during inflammatory diseases but also can cause or exacerbate pathological processes and result in further neurodegeneration. 

Increased thrombosis in the circulatory system would reasonably be expected to affect blood flow in small cerebral vessels, impairing exchange between the vessels and neurons and thus leading to vasculo-neuronal uncoupling. For example, it is known that TBI is associated with an almost immediate reduction in cerebral blood flow (CBF). It was reported that in the period of 2 h after a controlled cortical impact causing mild-to-moderate TBI, microthrombi occluded up to 70% of venules and 33% of arterioles [30].The reduced CBF seen in the traumatic penumbra caused by the formation of thrombi in the microcirculation [30] leads to secondary damages causing impairment in neuronal function and neurodegeneration, ultimately resulting in memory reduction [31].

The role of platelets in MS pathology has been speculated due to their interaction with leukocytes during their penetration of the BBB and the release of platelet-EC adhesion molecule-1 (PECAM-1) to the circulation [32]. In normal conditions, the basal expression of adhesion molecules is low; however, the expression of adhesion molecules on ECs and leucocytes is upregulated during inflammation [33]. Increased soluble PECAM-1 (sPECAM-1) is detected in the sera of MS patients [34]. The increased levels of sPECAM-1 may be a result of its increased release from microvessels and leukocytes during inflammation [34]. Platelet extravasation has been described in inflammatory reactions as a consequence of vascular rupture or increased permeability of undamaged venular endothelium by a transcellular route [35]. More commonly, platelets are described as “pathfinders” to direct leukocyte recruitment to the sites of their extravasation [36], and the significant role platelets play in the leukocyte recruitment into the inflamed brain microvessels was validated [37].

## 3. BBB Breakdown and Extravasation of Blood Cells

Leukocyte extravasation occurs primarily in post-capillary venules where shear stress is low [38]. The process of leukocyte migration from the blood stream to the extravascular space involves multiple steps. It begins with flowing leukocytes decelerating and slowly rolling on the activated endothelium [38,39], followed by adhesion strengthening and spreading, intravascular crawling, and finally, transcellular and/or paracellular transmigration [39]. Specific interactions of Fg with leukocytes and with ECs through its respective receptors on these cells, such as integrin αMβ2 and intercellular adhesion molecule-1 (ICAM-1), results in migration of leukocytes through ECs [40,41]. It has been shown that Fg dose dependently increases the adhesion of leukocytes to human umbilical vein ECs [42]. In an inflammatory condition, where Fg is elevated, it can be assumed that there is an increased adhesion of leukocytes on the luminal surface of ECs, leading to intraintimal accumulation and then extravasation of leukocytes. In fact, the finding of large depositions of Fg on the luminal surface of ECs in vivo represents a hallmark of certain inflammatory conditions, such as atherothrombosis [43]. Thus, it is possible that inflammation-induced elevation of the blood content of Fg potentially exacerbates the neuroinflammatory pathology.

Besides leukocytes, erythrocytes could also be found being extravasated during a breakdown of the BBB. However, if the vascular wall is not damaged significantly enough to allow penetration of red blood cells (RBCs), they do not cross the BBB even if the vessels are permeable to other cells or plasma components [44]. Extravasation of erythrocytes has been shown to cause oxidative injury to the brain [45]. It leads to the deposition of hemoglobin-derived neurotoxic products, including free iron. Decompartmentalization of iron from erythrocytes can cause brain edema and lipid peroxidation, leading to oxidative damages and neuronal death [45,46].

### Oxidative Stress in Cerebrovascular Disease

Under normal conditions, there is a balance between oxidant and antioxidant systems preventing oxidative damage. Oxidative stress develops when generation of reactive oxygen species (ROSs) is enhanced and/or ROS scavenging is impaired. Iron-derived ROSs are implicated in the pathogenesis of various vascular disorders, including vasculitis and reperfusion injury [47]. In the brain, ROSs have been shown to significantly alter BBB permeability and promote monocyte transmigration across the BBB. Therefore, any molecules that potentially generate enhanced ROSs potentially exacerbate neuroinflammation [48]. 

## 4. Increased Cerebrovascular Permeability and Neurodegeneration

### 4.1. Paracellular

Overall, changes in cerebrovascular permeability play the main role in the development of VCID. There are two modes of vascular permeability: paracellular (between the cells) [49] and transcellular (through the cells) [50,51,52]. Depending on the size of a substance, one or the other pathway can be used in extravasation. For example, a moderately large virus may not fit the gaps formed for a paracellular transport and would likely use a transcellular pathway involving vesicular transport [53]. In normal conditions, brain vessels are characterized with higher transendothelial electrical resistance than in peripheral circulation, indicating tighter junctions and therefore suggesting lesser paracellular transport than in skeletal muscle [54]. Similarly, much less transcellular transport (caveolar transport) occurs in brain vessels than in peripheral circulation [55]. During pathologies (inflammation), slight increases in these transport mechanisms can result in devastating consequences. These effects may result in enhanced water transport through paracellular and transcellular (via aquaporins) pathways and result in edema formation [56]. It is noteworthy that the physical breakdown of the BBB (rupture of vessels) that can occur during stroke or moderate to severe TBI and result in the accumulation of blood cells in the brain can result in changes of neuronal function and thus neurodegeneration [46]. Damage of vessels that leads to vascular rupture results in bleeding and the accumulation of blood components in the brain tissue. This process may not be considered a result of “vascular permeability” changes. Vascular permeability changes may occur in non-ruptured vessels that can be a result of alterations in the function of paracellular and/or transcellular transports. Altogether, changes in BBB integrity (vascular rupture) and/or permeability (enhanced paracellular and/or transcellular transports) inevitably lead to neurodegeneration and can result in memory reduction.

Chronic inflammation may also be one of the causes of enhanced cerebrovascular permeability. One of the indications of inflammation is a microvascular leakage of plasma substances and proteins and their deposition in the subendothelial matrix and interstitium [49]. We have shown a reduction in some endothelial junction proteins, along with increased endothelial layer permeability to albumin, caused by an elevated level of Fg, which is known to be associated with inflammation [57,58]. One of the possible mechanisms of increased paracellular transport can be explained by the findings that activity of inflammatory matrix metalloproteinases (MMPs) increases in many neurodegenerative diseases and after ischemic central nervous system (CNS) injury [59,60]. MMPs directly affect junction proteins and basement membrane extracellular matrix proteins [59]. Involvement of MMP-9 in increased extravascular deposition of Fg and an accompanied reduction in short-term memory has been found during TBI [61]. The levels of many adherence junctions and tight junction proteins are reduced in various neurodegenerative diseases, such as in AD and other diseases associated with dementia [44], amyotrophic lateral sclerosis [62], MS [63], and some animal models of neurodegeneration, such as aging [64]. 

### 4.2. Transcellular

Another pathway of cerebrovascular permeability, transcellular transport, includes caveolar transcytosis. While small molecules mainly use the paracellular pathway, high molecular weight proteins (e.g., Fg) cross the vessel wall mainly via caveolar transcytosis [52,65]. The main effect of this transport as opposed to the paracellular transport is that it can move relatively large proteins across the BBB. Crossing of the vascular wall for large proteins of the plasma via paracellular transport requires physically opening endothelial junctions wide enough to allow extravasation of these proteins. 

On the other hand, caveolae that can be ~30–80 nm in diameter [66,67,68], with the neck diameter reaching ~56 nm in ECs [69], can accommodate large proteins. For example, albumin and Fg, with their Stokes–Einstein radiuses of about 3.5 nm [70,71] and 8.4 nm [72], respectively, can easily fit into a caveola and be transported across the BBB.

Among the continuous endothelium found in many types of tissue, including lung, muscle, and brain, the ECs in the brain have more restricted permeability [73]. The critical characteristic of brain endothelium is that it establishes the barrier limits for the diffusion of blood-borne solutes and restricts molecular exchange [73]. These features include specialized tight junctions that restrict diffusion of molecules, a small number of endocytic vesicles, and lowered rates of transcytosis relative to peripheral vasculature [44]. Thus, in normal conditions, caveolar transcytosis is quite low and very little macromolecular cargo crosses the cerebral capillary endothelium [55]. However, during inflammation, activation of ECs results in enhanced caveolar transcytosis that can have devastating effects on brain cells [74,75,76].

Alterations in endothelial layer integrity, caveolar transcytosis, and the basal membrane result in the accumulation of high molecular weight proteins, normally found in plasma, in the extravascular space [77]. One such protein is Fg [77]. It is evident that deposition of Fg in the extravascular space of brain tissue during inflammatory diseases such as AD [78] and TBI [8,79] is associated with a decline in memory. Enhanced deposition of Fg results in favorable conditions for the formation of Fg-containing protein complexes such as Fg-amyloid beta (Aβ) [75,78,80] and Fg-cellular prion protein (PrP^C^) [81]. It has been shown that PrP^C^ can be endocytosed via caveolae [82]. We found that at an elevated level (e.g., during inflammation), Fg is transcytosed [74], is extravasated by caveolae [75], and then can directly interact with PrP^C^ [83]. It is known that, during neurodegenerative diseases, endogenous PrP^C^ undergoes a transformation to a conformationally altered scrapie prion protein (PrP^Sc^) that accumulates in the brain as insoluble aggregates [84]. The binding of Fg to PrP^Sc^ has also been documented [85]. It has also been found that PrP^C^ can bind readily to Aβ, indicating that it may act as a receptor that initiates a chain of events leading to neuronal destruction [86]. In addition, it has been shown that the specific interaction of Fg with Aβ [75,80] modifies Fg’s structure, leading to an abnormal fibrin clot formation more resistant to degradation [80,87]. Combined, these results suggest that a possible interaction of extravasated Fg with PrP^C^ and Aβ may result in the formation of aggregates highly resistant to degradation and lead to the neurodegeneration seen during neuroinflammatory diseases. In addition, the deposition of Fg and the formation of Fg-containing protein complexes in the extravascular space of the brain results in increased water transport and its accumulation in the interstitium, leading to the formation of edema and the resultant neurodegeneration [88]. 

## 5. Acute Phase Proteins in VCID

As a result of the development of systemic inflammation, blood plasma proteins such as albumin, Fg, C-reactive protein (CRP), and possibly some other high molecular weight acute reactant proteins may contribute to VCID. In response to injury and infection, at the expense of albumin synthesis, the liver enhances the synthesis of certain plasma proteins collectively known as acute phase proteins (APPs) [89]. The magnitude of the increase in the levels of these proteins varies. While CRP and serum amyloid A (SAA) can reach plasma levels of several hundred to a thousand-fold following acute inflammation, levels of haptoglobin and Fg do not increase more than two to tenfold [89,90]. Moreover, while the levels of CRP and SAA rapidly return to their normal range after the inflammation subsides, the levels of haptoglobin and Fg stay elevated for more than 21 days [90]. 

CRP is a 21 kD protein that has a similar structure to SAA [91]. Measuring the levels of CRP has been a standard of care in the clinic that can be a useful objective index to monitor the effectiveness of a therapy for a disease (inflammatory). At a normal level (0.8–9 μg/mL), CRP does not affect the BBB permeability [91]. On the other hand, it has been shown that when the level of CRP exceeds 2.5 μg/mL, it increases paracellular permeability of the BBB, affecting function but not the level of expression of tight junction proteins [91]. 

SAA is a small protein with a molecular weight of 12.5 kD that can be found in the blood of healthy individuals at the level of 20–50 μg/mL [92]. In subclinical inflammation, and for patients receiving glucocorticoid or immunosuppressive therapy, it has been suggested that SAA is a more sensitive biomarker than CRP [93]. It has been shown that Apo-SAA dose dependently increased the rat brain ECs permeability, shown by a significant reduction in transendothelial electrical resistance [94]. Interestingly, it has been shown that the circulating lipid free form of the SAA in the human plasma is 100 times lesser than that of SAA associated with high-density lipoprotein (HDL) [92]. Furthermore, the SAA-mediated impairment of the BBB was shown to be inhibited by the addition of HDL related to SAA in plasma [94]. Whether the free form of native SAA impairs the integrity of the BBB in pathological conditions remains unclear [94]. Given the SAA characteristic of high lipophilicity and the fact that most of the circulating SAA is associated with HDL, it is suggested that only a small amount of lipid-free SAA plays a major role in the BBB permeability changes compared to the other APPs. 

Haptoglobin is an acute phase glycoprotein that can be found in the serum of all mammals [95]. As it binds to free hemoglobin (Hb) with a high affinity, haptoglobin’s primary function is to facilitate Hb clearance. Hb is the prominent blood protein involved in transporting oxygen in the circulation. When unbound to haptoglobin and in the absence of other clearance mechanisms, free Hb can catalyze the formation of free radicals and mediate oxidative damages [95]. Haptoglobin is primarily synthesized in the liver. However, it has been shown that oligodendroglia can also synthesize haptoglobin, releasing it into the extracellular space, where it shows protective effects on brain cells from damages mediated by hemolytic product during intracerebral hemorrhage [96]. Haptoglobin production has also been described in other tissues, such as lung, skin, and kidney, during inflammatory conditions [97]. Although zonulin, a pre-haptoglobin precursor protein, has been shown to enhance small intestinal permeability, its direct effect in mediating BBB permeability has been questioned [98]. There are contrasting findings where preclinical in vitro data showed that zonulin potentially impaired BBB permeability [99], while other studies did not find evidence of its significant contribution in BBB permeability changes [98]. Taking into consideration that haptoglobin is found at a very low level in the normal brain [96] and that it has been shown to protect against Hb-induced toxicity [100], the prevailing role of haptoglobin can be considered to be neuroprotection. 

Fg is an acute phase reactant protein that is increased during inflammation [90]. The blood content of Fg increases not only during neuroinflammatory diseases such as AD [101], MS [102], TBI [103], or stroke) [104], but also during other inflammatory diseases such as cardiovascular diseases [105,106] and cancer [107]. It has been widely shown that Fg and its derivative fibrin are not only markers of inflammation [90], but also cause inflammatory responses [57,108,109,110,111]. The cause-and-effect relationship between elevated blood levels of Fg (HFg) and cardiovascular disease has been shown, and HFg is presumed to be more than just a byproduct of an inflammatory cardiovascular disease. It may independently or interactively modulate the severity and/or the progression of cardiovascular disease [105]. Fg deposition in brain parenchyma has been documented during conditions with an impaired BBB, such as MS [112]. In fact, extravascular deposition of Fg in the brain parenchyma seen in autopsy tissue samples of patients who suffered from MS is indicative of BBB impairment [113]. 

Changes in blood rheological properties that are caused by changes in blood viscosity, blood flow, RBC aggregation, leukocyte activity, and platelet thrombogenesis are associated with HFg. A significant role of Fg in blood viscosity changes has long been known [114,115,116,117]. In addition, it is established that Fg is directly involved in platelet thrombogenesis [118]. We have shown that HFg that occurs during hypertension (an inflammatory disease) enhanced development of platelet thrombogenesis [119,120]. Interaction of Fg and leukocytes during their activation has been well defined [42,121]. In addition, the role of Fg in increased RBC aggregation has been well established [122]. We have shown that a direct interaction of Fg with erythrocytes has a significant effect on RBC aggregation [123]. Furthermore, a direct correlation between blood viscosity and RBC aggregation during hypertension is well established [124,125]. Combined, these effects of Fg can easily result in development of hemostasis during inflammatory diseases and cause vasculo-neuronal uncoupling in the brain.

Specific interaction of Fg with the microvascular endothelium and the resultant vasoconstriction has been shown [126]. We have also shown that at elevated levels, Fg can activate ECs [111] and enhance caveolar transcytosis of proteins [127], resulting in the increased BBB permeability seen during TBI [128]. While out of vasculature, Fg can interact with and activate astrocytes [129,130] and, by directly interacting with neurons, generate (ROS), NO, and mitochondrial superoxide in these cells [83]. Moreover, as it is converted to fibrin in the extravascular space, it induces perivascular microglial clustering, promotes demyelination, and promotes dendrite and spine elimination in neurons, which has been shown to be associated with neurodegeneration and reduced neuronal density [131,132].

**Figure 1:**
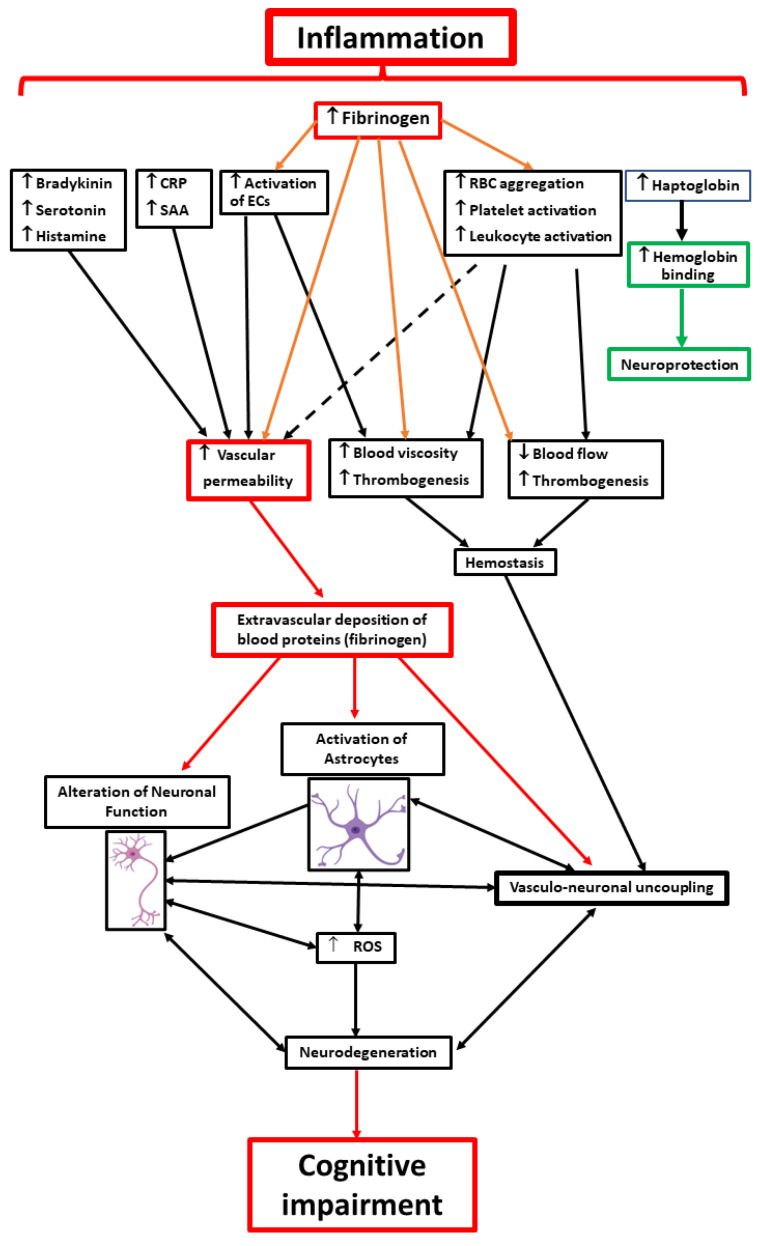
Schematic representation of the hypothesis of inflammation-induced vascular cognitive impairment. Among other factors that can affect vasculo-neuronal uncoupling, the factor that stands out is fibrinogen (Fg). It can be involved in changes of vascular permeability in various ways. Changes in vascular permeability result in direct activation of astrocytes and neurons through increased deposition of blood proteins (particularly of Fg) in the extravascular space, leading to neurodegeneration and a reduction in memory. Orange arrows indicate direct effects of Fg. Red arrows indicate direct effects. Dotted arrow indicates indirect effect. Green boxes and the arrow define anti-inflammatory pathway, while red boxes emphasize effects with strongest effects in VCID. Abbreviations: RBC—red blood cells, ECs—endothelial cells, CRP—C reactive protein, SAA—serum amyloid A, ROS—reactive oxygen species.

Although Fg is mainly synthesized and generated in the liver [133], in addition to being situated in plasma, it is accumulated in α-granules of platelets [134,135]. The release of Fg from α-granules [134] occurs slower than secretion from dense granules [136] and possibly as a second phase of platelet activation after content of the dense granules is released. Fg deposited on activated endothelial cells can become a binding site for even non-activated platelets via their surface receptor α_IIb_β_3_ [137]. It has been known that RBC aggregation can be promoted by several plasma proteins, such as Fg, α2-macroglobulin, and immunoglobulins M and G [138]. However, it was found that the only protein that had an effect on RBC aggregation on a biologically relevant level was Fg [138]. We have found that Fg had a specific interaction with RBCs via, most likely, integrin-like receptors on the surface of erythrocytes and promoted RBC aggregation [123]. Combined, these results suggest that during inflammation, when its blood content is elevated, Fg can be involved in platelet thrombogenesis and RBC aggregation, leading to blood flow reduction and decreasing supply to neural tissue with necessary nutrients, resulting in neurodegeneration. These findings suggest that Fg can be one of the most prominent agents in the circulatory system involved in vascular effects of neurodegeneration and memory reduction, i.e., in VCID. Some inflammation-induced and VCI-mediated mechanisms involved in memory decline are presented in Figure 1. 

## 6. Other Proteins Involved in VCID

It has been shown that Aβ is generated in both brain and peripheral tissues and is released into the circulatory system [139], where its level is correlated with increased risk of AD development [140,141,142]. Blood-derived Aβ can enter the brain tissue and cause neuronal dysfunction [143,144]. Strong association of Aβ peptide with Fg was linked to severity of AD [80]. A correlation of Aβ pathology and impairment in memory during TBI has been suggested [145]. In addition, there is evidence that links the occurrence of TBI to the onset and progression of AD and cognitive impairment [146]. Repetitive mild TBI has been shown to accelerate Aβ deposition, lipid peroxidation, and cognitive impairment in a transgenic mouse model of AD [146].

The extravasation of Aβ has a major role in the accumulation of Aβ in the CNS [147]. There is also evidence that Aβ accumulation itself affects brain vasculature and changes the function of the NVU [144]. It has been shown that the association of Fg and Aβ alters thrombosis [78] and results in the formation of clots with an abnormal structure and resistance to fibrinolysis [87]. Although the formation of complexes containing Fg/fibrin [148] and Aβ [149,150] is the hallmark of AD [78,80,148], some evidence indicates that the content of Aβ alone has a limited effect on memory [151,152]. These results suggest that formation of Fg–Aβ complexes can have a greater effect on memory reduction than the extravascular deposition of Fg or Aβ alone.

Although Aβ is strongly associated with AD [80], there is evidence of the greater role of cellular prion protein (PrP^C^) in memory reduction [151,152]. In addition, the role of PrP^C^ in TBI-associated memory reduction has been shown [153]. PrP^C^ is a cell surface, glycosylphosphatidylinositol anchored glycoprotein, abundantly expressed in neurons, glial cells [154], and endothelial cells [155]. It was shown that PrP^C^ participates in Aβ transcytosis through the BBB [156] and Aβ-mediated memory reduction during TBI [151]. We have recently shown that Fg can specifically interact with PrP^C^ on the surface of astrocytes [81,130]. Moreover, our data showed that Fg can form a complex with PrP^C^ in the extravascular space of mouse brains during mild-to-moderate TBI and was accompanied by short-term memory reduction [61]. TBI causes Aβ-PrP^C^-Fyn kinase activation, which also induces tau phosphorylation [153]. Fyn kinase is localized in the postsynaptic density of the brain, which is the primary site of signal transduction and processing, and its activity is linked to synaptic function [157,158]. 

It is well known that the deposition of Aβ plaques and tau-associated neurofibrillary tangles are a hallmark of AD. Both the deposition of amyloid and tau proteins have been implicated in the memory decline present during AD [159]. In fact, the direct interaction between the Aβ and specific regions of tau has recently been defined, suggesting that targeting only Aβ or only tau may not be the best treatment strategy during AD [160]. However, there are some conflicting data regarding effects of Aβ and tau on memory. Some data indicate that the content of tau, but not the levels of Aβ, in cerebrospinal fluid is associated with the severity of short-term memory impairment present in AD patients [161]. Other studies indicate that hyperphosphorylation of tau is not directly responsible for Aβ-induced neurodegeneration in vitro [162], and amyloid deposition has a greater association with microglial activation and memory reduction than tau pathology does [163]. Similarly, it has been shown that tau has a limited role in Aβ-induced memory impairment [164]. These results suggest that Aβ may have a greater effect than tau in memory reduction. This point can be substantiated by the fact that tau is exclusively present in nonvascular brain cells while Aβ, in addition to its presence in brain cells, can also be extravasated from the blood stream to further increase its overall content in the brain during neuroinflammation.

The neuropeptide substance P (SP) was first identified in the brain and gut in the early 1930s by Euler and Gaddum [165]. It is widely distributed in the central, peripheral, and enteric nervous systems and acts as a neurotransmitter and a neuromodulator that has a potent hypotensive property. It has been shown that during acute brain injury, SP was found perivascularly and linked to vasogenic edema formation [166]. It is suggested that SP plays a major role in secondary injury during neuroinflammatory diseases such as TBI. It has been found that SP mediates an increase in vascular permeability leading to the formation of edema [167]. Furthermore, SP itself may have a direct role in learning and memory, as it has been shown that blocking SP receptor expression in the hippocampus in the neostriatum impairs learning and memory in tested rats [168].

## 7. Some Other Inflammatory Agents Commonly Associated with BBB Disruption

Several other inflammatory mediators have been involved in modulation of BBB permeability. These are bradykinin, which increases BBB permeability by acting on B_2_ receptors, serotonin, which affects BBB permeability in some but not all studies, and histamine, one of the few CNS neurotransmitters consistently associated with BBB impairment [169]. Increased BBB permeability leads to the neurodegeneration and reduction in memory seen during diseases such as AD [144] and TBI [7,8,61,75].

## 8. Conclusions

In conclusion, we would like to emphasize that most of the systemic effects that are conveyed to neurons originate in the circulation. These effects exclude genetic, epigenetic, and some sensory effects that could directly affect neuronal function, which are not considered in the present review. Systemic alterations undeniably affect the composition of blood and properties of blood cells, plasma proteins, vascular cells, and/or vessels affecting blood flow. All these changes can positively influence the BBB integrity. As a result, any pathological alteration of the BBB property results in abnormal effects in glial and neuronal functions, eventually leading to possible neuroinflammation and neurodegeneration with high incidence of memory reduction. All these emphasize an imperative importance to study mechanisms of cerebrovascular permeability during various neurodegenerative diseases. Studying the link between BBB dysfunction and dementia might be key in finding the right window for intervention. To help accelerate the development of new and existing biomarkers for VCID, the MarkVCID consortium was formed under cooperative agreements with the NINDS and the National Institute on Aging in 2016 [170], involving multicenter studies whose mission is to identify and validate biomarkers for VCID. The overall goal of the consortium is to deliver high-quality biomarkers ready for use in clinical trials aimed at generating scientific breakthroughs in deeper understanding and treatment of VCID. New discoveries will not only open the possibility of restoring and/or maintaining properties of the intact BBB, but also will exploit ways for safe delivery of drugs through the BBB during various pathologies. Currently diagnosis for VCID is limited to clinical signs of dementia and/or magnetic resonance imaging, which may take place later in the course of the disease as a part of intervention or prevention. Therefore, developing biomarkers, preferably noninvasive markers, for early detection and prevention of VCID is an imperative goal for the future.

## Data Availability

Not applicable.

## Abbreviations

RBCs—red blood cells, ECs—endothelial cells, CRP—C reactive protein, SAA—serum amyloid A, ROSs—reactive oxygen species.
